# Glycosphingolipids within membrane contact sites influence their function as signaling hubs in neurodegenerative diseases

**DOI:** 10.1002/2211-5463.13605

**Published:** 2023-04-17

**Authors:** Jason Andrew Weesner, Ida Annunziata, Diantha van de Vlekkert, Alessandra d'Azzo

**Affiliations:** ^1^ Department of Genetics St. Jude Children's Research Hospital Memphis TN USA; ^2^ Compliance Office St. Jude Children's Research Hospital Memphis TN USA; ^3^ Department of Anatomy and Neurobiology, College of Graduate Health Sciences University of Tennessee Health Science Center Memphis TN USA

**Keywords:** endo‐lysosome, ER, glycosphingolipids, lysosomal storage diseases, membrane contact sites, mitochondria

## Abstract

Intracellular organelles carry out many of their functions by engaging in extensive interorganellar communication through specialized membrane contact sites (MCSs) formed where two organelles tether to each other or to the plasma membrane (PM) without fusing. In recent years, these ubiquitous membrane structures have emerged as central signaling hubs that control a multitude of cellular pathways, ranging from lipid metabolism/transport to the exchange of metabolites and ions (i.e., Ca^2+^), and general organellar biogenesis. The functional crosstalk between juxtaposed membranes at MCSs relies on a defined composite of proteins and lipids that populate these microdomains in a dynamic fashion. This is particularly important in the nervous system, where alterations in the composition of MCSs have been shown to affect their functions and have been implicated in the pathogenesis of neurodegenerative diseases. In this review, we focus on the MCSs that are formed by the tethering of the endoplasmic reticulum (ER) to the mitochondria, the ER to the endo‐lysosomes and the mitochondria to the lysosomes. We highlight how glycosphingolipids that are aberrantly processed/degraded and accumulate ectopically in intracellular membranes and the PM change the topology of MCSs, disrupting signaling pathways that lead to neuronal demise and neurodegeneration. In particular, we focus on neurodegenerative lysosomal storage diseases linked to altered glycosphingolipid catabolism.

## Graphical abstract

This review focuses on the impact of glycosphingolipids (GSLs), clustered within specific membrane contact sites (MCSs), on their protein and lipid compositions and function. Alterations in GSL levels at MCSs due to their abnormal catabolism affect their structural characteristics and signaling properties, leading to neurodegeneration in the lysosomal storage diseases: Gaucher disease, GM1‐gangliosidosis and Niemann‐Pick Type C.

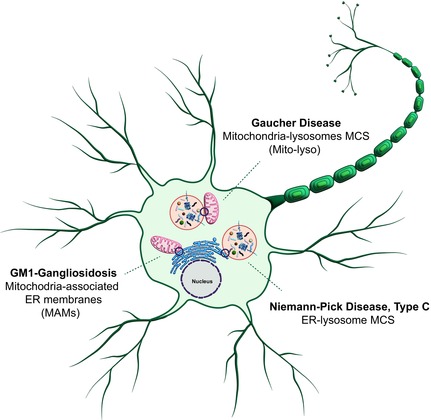

**Abbreviations**
AD: Alzheimer's disease
ALS: amyotrophic lateral sclerosis
ATP: adenosine triphosphate
b‐GAL: beta‐galactosidase
CA^2+^
: calcium
CBE: conduritol‐B epoxide
ER: endoplasmic reticulum
FFAT: Phe‐Phe‐ in an acidic tract
Fis1: fission protein 1, mitochondrial
FTD: frontotemporal dementia
GBA1: β‐glucocerebrosidase
Gcase: β‐glucocerebrosidase
GD: Gaucher disease
GDP: guanosine diphosphate
GEMs: GSL‐enriched micromembranes
GlcCer: glucosylceramide
GM1: GM1‐ganglioside
GRAMD1b: GRAM domain containing 1b
GRP75: glucose‐regulated protein 75
GSLs: glycosphingolipids
GTP: guanosine‐5′‐triphophate
IP3: inositol 1,4,5‐triphosphate
IP3R1: inositol 1,4,5‐triphosphate receptor 1
LSD: lysosomal storage disease
MAMs: mitochondria‐associated ER membranes
MCSs: membrane contact sites
MSP: major sperm protein
mTORC1: mammalian target of rapamycin complex 1
NPC: Niemann‐Pick disease, type C
NPC1: Niemann‐Pick disease type C protein 1
NPC2: Niemann‐Pick disease type C protein 2
OMM: outer mitochondrial membrane
ORP1L: oxysterol‐binding protein‐related protein 1
ORP5: oxysterol‐binding related protein 5
OSBP: oxysterol‐binding protein
PD: Parkinson's disease
PI4P: phosphatidylinositol 4‐phosphate
PM: plasma membrane
PTPIP51: protein tyrosine phosphatase‐interacting protein 51
Rab7: Ras‐related protein 7
siRNA: small interfering ribonucleic acid
SNX2: sorting nexin‐2
SOCE: store operated calcium entry
STARD3: steroidogenic acute regulatory protein (StAR)‐related lipid transfer (START) domain 3
STARD3NL: STARD3 N‐terminal like protein
STIM1: stromal interaction molecule 1
TBC1D15: Tre2/Bub2/Cdc16 (TBC1)‐domain family member 15
TMEM192: transmembrane 192
TPC2: two pore segment channel 2
TRPML: transient receptor potential cation channel, mucolipin subfamily
UPR: unfolded protein response
VAMP: vesicle‐associated membrane protein
VAP: VAMP‐associated protein
VDAC‐1: voltage‐dependent anion channel protein 1
VPS13: vacuolar protein sorting‐associated protein 13

The physiology of all eukaryotic cells is based on the coordinated functions of a complex network of intracellular compartments, defined by membrane‐enclosed organelles that communicate with each other and with the plasma membrane (PM) [1, 2]. Each organelle provides a unique luminal environment and membrane composition that have both evolved to cope with a myriad of cellular functions [1, 2]. Organelles can sense the metabolic state of each other in response to specific physiological or pathological stimuli. They also cooperate when different steps of biochemical reactions/processes take place in more than one compartment and require the selective exchange of ions and other molecules or metabolites between membranes. Therefore, organellar membranes must maintain a highly dynamic structure that can undergo rapid changes in its lipid and protein composition, depending on the functional state of the cell [3]. In particular, the lipid content dictates the physical properties of membranes and modulates the functions of neighboring membrane proteins and protein complexes [1, 4, 5]. Understanding of how these lipid components affect membrane physiology remains a challenge, although, progress has been made through the use of advanced biochemical and imaging methods applied to the study of membrane‐associated processes in human diseases, particularly neurodegenerative diseases.

Adding to the challenge is the evidence that the PM and organellar membranes are organized into discrete microdomains, which are formed by the selective assembly of specific lipids and proteins [6, 7]. An example of such microdomains are the lipid rafts, which are enriched in sphingomyelin, cholesterol, glycosphingolipids (GSLs), and saturated phospholipids and are found primarily in PMs but also in intracellular membranes [8, 9]. A similar composition is shared by membrane regions where GSLs cluster, known as the GSL‐enriched microdomains, or GEMs, which are particularly abundant in GSL‐rich cells, such as neurons [6, 10].

## Membrane contact sites

When membrane microdomains within distinct organelles, or the PM, come in close proximity (~ 10–50 nm) to each other without fusing, they form membrane contact sites (MCSs) [11, 12]. These specialized structures, present in virtually all cell types, add a tier to the regulated communication between the juxtaposed membranes, allowing for rapid and efficient responses to several physiological or pathological changes [12, 13]. Tethering of membranes at MCSs is transient and creates dedicated “hit and run” signaling hubs that coordinate lipid synthesis and transport across membranes, exchange of metabolites and ions (e.g., Ca^2+^), organellar dynamics through fission and fusion events, and other metabolic processes that do not require vesicular trafficking [13, 14, 15, 16].

**Fig. 1:**
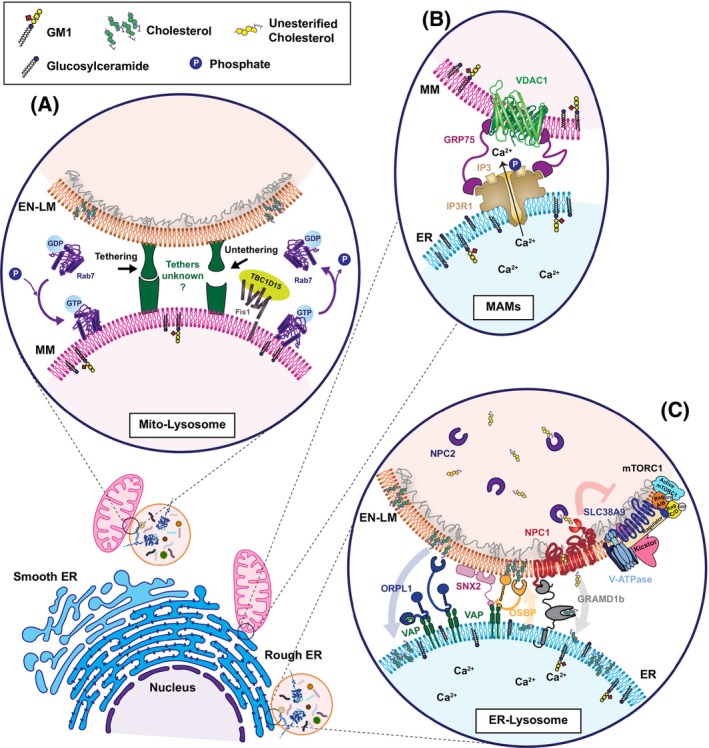
Membrane contact sites in LSDs: Physiological tethering complexes in (A) mitochondria‐lysosome MCSs, (B) mitochondria‐associated ER membranes and (C) ER‐lysosome MCSs that are dysregulated in LSDs. GlcCer can be found luminal or cytosolic within the ER membranes while all higher order GSLs are only found luminal in the organelles. GDP, guanosine diphosphate; GTP, guanosine‐5′‐triphophate; RAB7, Ras‐related protein 7; FIS1, mitochondrial fission 1 protein; TBC1D15, Tre2/Bub2/Cdc16 (TBC1)‐domain family member 15; VDAC‐1, voltage‐dependent anion channel protein 1; GRP75, glucose‐regulated protein 75; Ca^2+^, calcium; IP3, inositol 1,4,5‐triphosphate; IP3R1, inositol 1,4,5‐triphosphate receptor 1; NPC1, Niemann‐Pick disease, type C1; NPC2, Niemann‐Pick disease, type C2; mTORC1, mammalian target of rapamycin complex 1; Slc38A9, solute carrier family 38 member 9; V‐ATPase, vacuolar‐type ATPase; ORP1L, oxysterol‐binding protein‐related protein 1; VAP, VAMP (vesicle‐associated membrane protein)‐associated protein; SNX2, sorting nexin‐2; OSBP, oxysterol‐binding protein; GramD1B, GRAM domain containing 1B; Rag A/B, ras‐related GTP binding A/B; Rag C/D, ras‐related GTP binding C/D; Ragulator, pentameric complex (p18, p14, MP1, c7orf59, HBXIP); Kicstor, complex (KPTN, ITFG2, C12orf66, SZT2).

Considering the size and widespread structure of the endoplasmic reticulum (ER) membranes, it is not surprising that this organelle functions as the “master in command” of MCSs, physically engaging with all other cellular membranes, such as those of the mitochondria (known as mitochondria‐associated ER membranes, or MAMs), Golgi apparatus, endosomes, lysosomes, peroxisomes, lipid droplets, and the PM (known as ER‐PM junctions) (Fig. 1) [13, 17, 18, 19, 20, 21]. These connections *in trans* allow the ER to maintain homeostatic control over the biosynthetic, secretory, and endocytic pathways [20, 22]. However, MCSs are not just a prerogative of the ER; other intracellular organelles, such as mitochondria and endosomes/lysosomes, form MCSs either with each other (mito‐lyso MCSs), or with peroxisomes, lipid droplets, and the PM (Fig. 1) [23, 24, 25, 26].

The diversity of cellular functions that have now been ascribed to MCSs requires specific arrangements of protein and lipid components that cluster or disperse in a dynamic manner at either of the apposing membranes [14, 27]. Lipids, such as cholesterol, phospholipids, and glycolipids, particularly GSLs, have been shown to drive the partitioning of membrane proteins (i.e., transporters, receptors, or channels) within MCSs to modulate signaling events, many of which involve lipid metabolism and Ca^2+^ signaling [6, 28, 29, 30]. These two processes are fundamental for the nervous system, where they are often interdependent [31, 32]. Several lipids and proteins residing within MCSs function as tethers, bridging the two apposing membranes while maintaining the functional distance between them [18, 33, 34]. Some of these tethers are conserved among species and are integral constituents of multiple MCSs. A good example is the family of ubiquitous tethering proteins known as vesicle‐associated membrane protein (VAMP)‐associated proteins (VAPs) that are anchored to ER membranes via their C‐terminal tail and are present in virtually all MCSs formed between the ER and other organelles [19, 20, 34]. Through a specific major sperm protein (MSP) sequence domain at their N‐termini, the VAPs bind various lipid transfer proteins that embed the so called Phe‐Phe‐ in an acidic tract (FFAT) motif, which can interact *in trans* to the juxtaposed membranes [17, 19, 20]. This configuration not only facilitates the formation of MCSs but also permits the transfer of phospholipids and, in some cases, cholesterol and ceramide, between organelles. The extent to which the lipid components impact the properties of MCSs has recently been highlighted in an elegant study showing that ER membranes can undergo phase separation into lipid microdomains that sort proteins within MCSs, thus modulating membrane fluidity at contact sites [35].

These observations reinforce the notion that alterations in the lipid makeup of MCSs can drastically change their structural and functional characteristics and impinge on their ability to respond to cellular and environmental cues, ultimately leading to cell demise and disease, particularly those of the nervous system.

### Mitochondria‐associated ER membranes

The MAMs were the first MCSs visualized in the 1950s by electron microscopy of rat liver cells and described as electron‐dense structures formed by close juxtaposition of the ER and the mitochondrial membranes [11]. These structures were later found across many organisms and cell types, but their functional implications were not revealed until the early nineties when Vance and coworkers found that MAMs were the site of phospholipids biosynthesis and transfer between the ER and the mitochondria [36, 37]. Since these pioneering studies, many other cellular processes have been assigned to these MCSs, including fission/fusion events that control mitochondrial shape and size, ER‐mitochondria‐mediated apoptosis, mitochondrial energy metabolism, autophagy induced by mitochondrial energy stress and, importantly, regulation of Ca^2+^ flux between the ER and mitochondria [6, 16, 21, 38, 39, 40].

Innumerable ER and mitochondrial proteins have been found to colocalize within the MAMs, many of which function as *bona fide* tethers, and directly, or indirectly, orchestrate membrane lipid shuttling and Ca^2+^ transfer between the organelles [38, 40]. These include the complex between VAPB, at the ER side, and the outer mitochondrial membrane (OMM)‐resident protein tyrosine phosphatase‐interacting protein 51 (PTPIP51), as well as the complex formed by ER‐resident inositol 1,4,5‐triphosphate (IP3)‐sensitive Ca^2+^ channel (IP3R‐1), and the mitochondrial proteins voltage‐dependent anion channel‐1 (VDAC‐1) and glucose‐regulated protein 75 (GRP75) (Fig. 1B) [41, 42]. At the MAMs, PTPIP51 binds and transfers phosphatidic acid from the ER to mitochondria in a process needed for the synthesis of cardiolipin, an essential phospholipid found in the inner mitochondrial membrane [43]. In addition, although not directly involved in Ca^2+^ transfer, the VAPB‐PTPIP51 complex facilitates the uptake of Ca^2^ released by the IP3R‐1/GRP75/VDAC‐1 complex into the mitochondria during autophagosome formation and synaptic activity [44]. IP3R‐1/GRP75/VDAC‐1 forms a Ca^2+^ megapore that localizes to the MAMs and clears Ca^2+^ at the mouth of IP3R‐1 channel to regulate the kinetics of Ca^2+^ release from the ER [28, 42].

Following the discovery of MAMs, another MCS was identified between the ER membranes and the PM, named the ER–PM junctions [45]. These contact sites share structural and functional characteristics with the MAMs, implying a strict interplay between these cellular compartments. Indeed, similar to MAMs, the ER–PM junctions have two major tasks: coordinate Ca^2+^ dynamics, particularly in response to depletion of ER Ca^2+^ stores, and regulate lipid transfer/shuttling between the ER and PM, especially phospholipids and sterols [18, 46]. These cellular processes have since been documented in all MCSs so far identified that involve the ER membranes [15, 20, 47].

### ER‐endosomes/lysosomes

The ER forms contact sites with early and late endosomes, as well as lysosomes [19, 48, 49]. Several tethering proteins that facilitate the establishment of these MCSs have a role in cholesterol/phospholipid transport and in positioning endo‐lysosomes within the cell cytoplasm [19, 48, 49, 50, 51, 52]. For instance, VAPA and VAPB on the ER membranes have been shown to interact with several endosomal proteins: oxysterol‐binding protein (OSBP), OSBP‐related protein 1L (ORP1L), steroidogenic acute regulatory protein (StAR)‐related Lipid Transfer (START) domain 3 (STARD3), STARD3 N‐terminal like protein (STARD3NL), and sorting nexin 2 (SNX2) [19, 48, 49, 51, 52, 53, 54]. OSBP was originally described as a VAP tether at ER‐Golgi contact sites, where it transports cholesterol in exchange for phosphatidylinositol 4‐phosphate (PI4P) [55]. However, OSBP was also found to localize to ER‐lysosome MCSs, where it exerts its lipid transport properties to move cholesterol from the ER to the lysosomes (Fig. 1C) [49]. ORP1L is a cholesterol‐binding protein that, under low cholesterol concentration, undergoes conformational changes that allow it to bind to VAPA, promoting the formation of ER‐endosome contact sites (Fig. 1C) [52]. ORP1L can also interact with the small GTPase, Rab7, present in the membrane microdomains of late endosomes/lysosomes marked by the cholesterol transporter transmembrane protein, Niemann‐Pick Type C protein 1 (NPC1) [52]. NPC1 is now recognized as an integral component of these contacts sites, where it egresses cholesterol from the lysosomes to the ER by associating with oxysterol‐binding related protein 5 (ORP5) [48, 56]. STARD3 is another late endosome, multidomain membrane protein involved in cholesterol transport [53, 54]. This protein forms a complex with its cognate protein STARD3NL and VAPA/B, tethering the two organelles together [54]. Lastly, the component of the retromer complex, SNX2, is a phosphoinositide binding protein that also interacts with VAPA/B and tethers the ER to the endosomes. At these MCSs, the VAP‐SNX2 interaction controls the endosomal pool of PI4P, which is regulated by OSBP [19]. These few examples are indicative of the involvement of tethers at the ER‐endosome and/or ER‐lysosome MCSs in securing the connection between the organelles and the regulated transfer of lipids between their apposing membranes. It remains to be elucidated whether different tethers have distinct tasks depending on the status of the cells or cell type, particularly in the nervous system, and whether GSLs participate in these functional MCSs.

Recent studies have implicated ER‐lysosome MCSs in the regulation of mechanistic target of rapamycin complex 1 (mTORC1), a central regulator of cell metabolism and growth [49, 57]. These authors have shown that the positioning of mTORC1 at the lysosomal membrane and the activation of mTORC1 signaling are influenced by the cholesterol concentration in the ER‐lysosome MCSs, which is controlled by the activity of OSBP on the lysosomal limiting membrane (Fig. 1C) [49]. The opposite function is exerted by GRAM domain containing 1b (GRAMD1b) tethered to NPC1, which exports cholesterol from late endosomes/lysosomes to other organellar membranes, including those of the ER (Fig. 1C) [48].

In addition to cholesterol shuttling, the ER‐lysosome MCSs regulate Ca^2+^ flux between the two organelles [47]. In this setting, lysosomal Ca^2+^ intake can have two downstream effects: It can amplify cytosolic Ca^2+^ signaling evoked by the activation of lysosomal Ca^2+^ channels such as transient receptor potential cation channel, mucolipin subfamily (TRPML) or two pore segment channel 2 (TPC2); it can also attenuate the effects of cytosolic Ca^2+^ signaling by rapidly sequestering Ca^2+^ released by the ER stores [58]. Imbalanced Ca^2+^ internalization/release at ER‐lysosome MCSs has a direct impact on endo‐lysosomal functions, including fusion and fission, lysosomal movement/positioning and autophagy [58, 59].

### Mitochondria‐lysosomes

Membrane contact sites between mitochondria and lysosomes (mito‐lyso) were identified in 2017, when investigators observed the juxtaposition of these organellar membranes by super resolution light and electron microscopy of live cells [60, 61, 62]. These contact sites are formed by tethering events that are distinct from the membrane interactions between the organelles that mediate mitochondrial degradation by the lysosomes, through the process of mitophagy [63]. Bidirectional communication in mito‐lyso MCSs has now emerged as a key pathway to maintain cell homeostasis [23]. In particular, it has been shown that the dysregulation of these contact sites contributes to neuropathogenesis in adult and pediatric neurodegenerative diseases [64, 65, 66].

Although exact tethering complexes of mito‐lyso MCSs have not yet been uncovered, lysosomal tethering and transport at these contact sites appear to be dependent on and regulated by Rab7 that is recruited to the lysosomal compartment in its active GTP‐bound state (Fig. 1A) [60]. Contact formation is promoted by binding of GTP‐Rab7 to effector proteins within the lysosomal membrane, which most likely represent *bona fide* tethering molecules of these MCSs. On the contrary, it was recently demonstrated in rat liver and cultured cells that the untethering of mito‐lyso contacts is mediated by the mitochondrial resident protein, fission protein 1 (Fis1), which recruits the Rab7‐GTPase activating protein, Tre2/Bub2/Cdc16 (TBC1)‐domain family member 15 (TBC1D15), to the OMM, resulting in Rab7‐GTP hydrolysis and loosening of the two organellar membranes (Fig. 1A) [60, 67]. In addition to participating in mito‐lyso tethering/untethering processes, Rab7‐GTP hydrolysis has also been implicated in mitochondrial fission regulated by TBC1D15 within these MCSs [60, 68]. Furthermore, the STARD3 ER‐lysosome tether appears to be involved in cholesterol transport also across mitochondrial and endo‐lysosomal membranes, likely occurring within mito‐lyso MCSs [69]. These observations point to STARD3 as a potential tether of the mito‐lyso MCSs and reiterate the multitasking activity of the same tethers at different MCSs. Interestingly, a recent publication describes ORP1L‐mediated recruitment of Rab7 to form triple contact sites involving ER, lysosomes and mitochondria [51]. These authors propose that ORP1L at these specialized MCSs transport phosphatidylinositides between endo‐lysosomes and mitochondria [51]. Lastly, as it is the case for other contact sites, mito‐lyso MCSs have also been recognized as the site of Ca^2+^ flux between these two organelles mediated by the lysosomal Ca^2+^ channel, TRPML1, whose loss of function is the primary cause of the lysosomal storage disease (LSD), mucolipidosis type IV [70].

## MCSs and neurodegeneration

In recent years, numerous studies have implicated components of MCSs in the pathogenesis of common adult neurodegenerative diseases associated with aging, including Alzheimer's Disease (AD), Parkinson's Disease (PD), Frontotemporal Dementia (FTD), and Amyotrophic Lateral Sclerosis (ALS), to mention a few [20, 23, 40, 64, 71, 72, 73, 74]. In these conditions, MCS dysfunction has either been linked to mutations that directly affect one of the tethering proteins or to altered activity of nontether components of the MCSs. Given that this subject has been extensively reviewed in several recent publications [20, 23, 75, 76, 77, 78, 79], here we cite only those examples that have been directly or indirectly linked to defects in tethering complexes functioning as lipid transporters at MCSs. For example, several genetic mutations affecting the activity of the VAPB isoform in MCSs (specifically those formed with ER membranes) appear to disrupt phospholipid homeostasis and Ca^2+^ signaling, and to activate the ER stress response in a familial form of ALS, known as ALS type 8 [75]. In addition to these *VAPB* mutations, perturbation of the tethering complex VAPB‐PTPIP51 has also been clinically linked to PD, ALS and FTD [71, 72, 73, 74, 80]. These groups demonstrated that in PD neurons α‐synuclein aggregation within the MAMs disrupts VAPB‐PTPIP51 tethering and loosens ER‐mitochondria contacts, leading to autophagosome formation, altered mitochondrial Ca^2+^ uptake, and impaired ATP synthesis [80, 81].

Besides these pathogenic events linked to VAPB‐PTPIP51 dysfunction at MCSs, recent findings showed that under physiological conditions this complex promotes MAM formation at the synapses, and in such capacity stimulates neuronal activity [44]. In fact, downregulation of either protein by silencing their expression in cultured hippocampal neurons affects synaptic function and dendritic spine morphology [44]. These studies squarely implicate the VAP tethering proteins in maintaining neuronal homeostasis and imply that damage to ER‐mitochondria signaling at the MAMs contributes to synaptic dysfunction, which is again a prominent pathological feature of PD, ALS and FTD [44, 74].

Another family of VAP‐interacting proteins, whose mutations have been linked to neurodegenerative diseases, is the lipid transporter vacuolar protein sorting‐associated protein 13 (VPS13) [20]. These proteins interact with VAP members on the ER membrane, tethering them to mitochondria (VPS13A), endosomes/lysosomes (VPS13C), lipid droplets (VPS13A and C), and peroxisomes (VPS13D) [20, 82]. Mutations in each of the 4 *VPS13* paralogues are associated with distinct neurological disorders, such as chorea acanthocytosis (*VPS13A*), Cohens syndrome (*VPS13B*), early onset PD (*VPS13C*), and a newly identified spastic ataxia (*VPS13D*) [20]. Although in these cases, the underlying molecular pathways have not been identified, disruption of lipids' transport at MCSs and mitochondrial defects have been proposed as two of the most likely pathogenic mechanisms [20].

These selected examples reiterate the role played by the lipid components of MCSs in maintaining their homeostatic control over normal processes in the nervous system. However, most of these studies have focused on how phospholipids and cholesterol within MCSs are involved in the pathogenic cascade that contributes to neurodegeneration. Much less attention has been paid to the involvement of glycolipids, particularly GSLs, in the regulation of neurodegenerative processes in MCSs. In the following sections, we discuss how altered GSL content and distribution within MCSs affect cellular proteostasis and drive neurodegeneration in a group of pediatric neurodegenerative diseases, the LSDs, caused by aberrant lysosomal catabolism and primary or secondary accumulation of GSLs [83].

## Lysosomes – LSD

The lysosomal system comprises an array of heterogeneous, acidic organelles that are essential for cell physiology and homeostasis. Their biogenesis is transcriptionally and epigenetically regulated, depending on the cell types and the metabolic status of the cells. The primary task of lysosomes is the catabolism of long‐lived macromolecular substrates, which reach the organelles via the biosynthetic, endocytic, autophagic or phagocytic rout, and the recycling of their breakdown products [84]. These basic lysosomal processes are achieved by the coordinated activities of a battery of intraluminal hydrolytic enzymes, membrane proteins or protein complexes, ion channels, transporters, solute carriers and a multi‐subunit vacuolar H^+^ ATPase pump [83, 84]. Once dismissed as the dead end of the cell requiring little or no regulation, these organelles have emerged as an essential metabolic center that control nutrient sensing, amino acid and ion homeostasis and Ca^2+^ signaling, among other functions [84]. They also have the capacity to respond to physiological and pathological cues by changing their number, cellular position, and specific protein and lipid contents [84]. As mentioned earlier, lysosomes are in dynamic communication with each other as well as with other intracellular compartments, and the cell exterior, forming functional MCSs. The broad spectrum of macromolecular cargos that converge to the lysosomal system reflects the diversity of biological processes that these organelles control, including autophagy, phagocytosis, endocytosis/exocytosis, plasma membrane repair, cholesterol trafficking, pathogen resistance, cell signaling, and cell death [84].

### Lysosomal storage disease

Dysfunction of the lysosomal system defines a large group of over 70 inborn errors of metabolism known as LSDs [83, 85]. These catastrophic conditions are genetically and clinically heterogenous, affecting multiple organs and the nervous system, so collectively they make up the highest number of pediatric neurodegenerative diseases [83, 85]. LSDs are caused by mutations in genes encoding lysosomal glycosidases, proteases, integral membrane proteins, transporters, enzyme modifiers or activators, although the majority are linked to deficiency of a single glycosidase [83, 85]. These enzymes are responsible for the stepwise cleavage of sugar residues from the glycan chains of glycoconjugates, such as GSLs. Loss of function of any of these enzymes or other lysosomal constituents results in impaired processing/degradation of macromolecular substrates, abnormal transport of lipids and metabolites across the lysosomal membrane, and primary or secondary storage of undigested or partially processed compounds in lysosomes and other cell compartments. These combined events evoke a sequela of pathological manifestations that vary depending on the cells/tissues and their physiological state, and are mostly attributable to the biochemical properties, cellular distribution and type of storage material, which ultimately lead to organ dysfunction and degeneration [83, 85].

The complex pathophysiology of LSDs is likely the result of multiple deregulated pathways (i.e., autophagy, endocytosis, phagocytosis lysosomal exocytosis, oxidative stress, inflammatory and innate immune responses, and cell death) that coalesce during the course of the disease, each contributing to specific pathological aspects and clinical outcome [83, 85]. Depending on the type of disorder, the age of onset, and the severity of clinical symptoms, LSD patients are classified as congenital/early infantile, late infantile, or juvenile/adult types. However, they mostly present with a continuum of disease severity, which is also influenced by the patient's genetic and epigenetic makeup, as well as dietary and environmental factors. In general, mutations that completely abrogate the enzyme catalytic activity are associated with the most severe neurodegenerative phenotypes [83, 85].

Notably, pediatric neurodegenerative LSDs often display neuropathological features of more prevalent adult conditions associated with aging, including neurodegenerative diseases such as AD and PD: motor weakness, developmental delay, acroparesthesia, psychiatric and behavioral deficits, seizures, myoclonus epilepsy, neuropathy, neurodegeneration, and chronic neuroinflammation are recurrent neurological signs in LSD patients [83, 85]. It is therefore understandable that deregulated pathways underlying neuropathogenesis in LSDs are often shared by adult neurodegenerative diseases; hence, targeting these pathways could pave the way for potential common therapeutic modalities for both groups of diseases.

Relevant for this review are the lipid storage diseases, a subgroup of LSDs that include the glycosphingolipidoses, which are associated with progressive accumulation of GSLs in intracellular membranes, particularly neuronal membranes [86]. Faithful animal models of LSDs have proven ideal tools to elucidate how aberrant levels/distribution of GSLs at specific MCSs modify their topology and functional characteristics and initiate a pathogenic cascade that contributes to neurodegeneration [28, 48, 49, 64, 86]. Here, we focus on the only three examples of LSDs that have been directly linked to MCSs dysfunction so far: GM1‐gangliosidosis, Niemann‐Pick disease, type C (NPC), and Gaucher disease (GD), where neuropathogenic pathways have been attributed to MCSs dysfunction due to the accumulation of GSLs or cholesterol. However, we predict that many of these pathways may be common to other GSL storage diseases, as well as adult neurological disorders.

## GM1‐gangliosidosis and MAM's dysfunction

GM1 ganglioside (GM1) is a sialylated GSL abundant in the adult mammalian brain, accounting for 10–20% of the total ganglioside pool of neuronal PMs (reviewed by Sonnino in this issue) [87]. Aberrant GM1 catabolism is associated with the GSL storage disease GM1‐gangliosidosis [88, 89], a neurosomatic disorder caused by genetic deficiency of lysosomal β‐galactosidase (β‐GAL). This enzyme, encoded by the *GLB1* gene, is solely responsible for the breakdown of GM1 in lysosomes [88, 89]. Patients with GM1‐gangliosidosis develop a systemic condition with onset of clinical symptoms between infancy and adulthood, and include severe growth retardation, developmental delay, multiple organ dysfunction, progressive and generalized neurodegeneration, psychomotor retardation, ataxia, slowly progressive dementia and parkinsonian features. The neuropathological outcome of GM1‐gangliosidosis is likely the consequence of the relentless buildup of GM1 in the nervous system downstream of loss of β‐GAL activity [88, 89]. Notably, a number of studies have shown the occurrence of Aβ‐generating secretases in GM1‐containing lipid rafts, and the presence of Aβ peptides associated with GM1 in a postmortem brain from a patient with early‐stage AD [83]. Moreover, adult cases of GM1‐gangliosidosis develop prominent parkinsonian signs [90]. Thus, although no *GLB1* mutations or polymorphisms have yet been liked to increased risk of AD or PD, there are clear parallels between these neurodegenerative diseases.

Both small and large laboratory animal models of GM1‐gangliosidosis have been used to investigate disease pathogenesis and to test experimental therapies [88, 89]. The first and most extensively studied is a *β‐Gal* knockout mouse (*β‐Gal* ^*−/−*^) that recapitulates the early onset forms of the human disease [91]. Deficient mice develop a range of neurological signs, including tremor, ataxia, paralysis of the hind limbs, and stiff tail, likely due to an autoimmune response [91]. Widespread lysosomal vacuolation of cells in the brain and spinal cord is accompanied by progressive lysosomal accumulation of GM1, which eventually leads to massive loss of neurons and neuroinflammation [92, 93].

At the molecular levels, impaired degradation of GM1 in lysosomes of *β‐Gal* ^*−/−*^ neurons results in the abnormal buildup of this ganglioside in internal membranes, in particular those of the ER [28, 88, 89]. Here, GM1 initiates a pathogenic cascade, that begins with the depletion of the ER Ca^2+^ stores and activation of the unfolded protein response, and culminates in an unfolded protein response (UPR)‐mediated apoptosis [94]. The Ca^2+^ released from the ER is buffered by the mitochondria to dampen the increased cytosolic Ca^2+^ concentration. In this case, high levels of GM1 at the ER membranes favor the physical juxtaposition between ER and mitochondria and, in turn, the formation of the MAMs [28]. This finding identified GM1 as a normal constituent of the MAMs, and in particular of the GEM fraction of these MCSs [28]. Within the GEMs/MAMs, GM1 interacts with the phosphorylated form of the IP3R‐1 Ca^2+^ channel (P‐IP3R1), maintaining it active at the opening of the IP3R‐1/GRP75/VDAC‐1 Ca^2+^ megapore. This protein configuration, coupled to an increased number of MAMs, drives a continuous flux of Ca^2+^ from the ER to the mitochondria, ultimately leading to mitochondrial Ca^2+^ overload, and activation of the mitochondrial leg of the apoptotic pathway [28]. These pathogenic mechanisms in *β‐gal* ^*−/−*^ neurons squarely link GM1 accumulation at the MAMs to neuronal cell death in GM1‐gangliosidosis.

The question that remains is how GM1 is sequestered to the internal membranes of *β‐gal* ^*−/−*^ neurons. Given that most MCSs have lipid transport capacity, a potential mechanism of GM1 translocation to the ER and mitochondrial membranes is through ER‐PM junctions and/or ER‐lysosome MCSs.

## NPC and ER‐lysosome dysfunction

Niemann‐Pick disease, type C is a lipid storage disorder caused by the deficiency of one of two proteins, NPC1 (*NPC1* gene) or NPC2 (*NPC2* gene), although *NPC1* mutations are prevalent in the patient population [83, 95]. NPC1 is a lysosomal transmembrane protein that functions as a cholesterol transporter, while NPC2 is a soluble cholesterol‐binding protein. Although NPC1 and 2 have distinct biochemical properties and lysosomal localization, mutations affecting their functions result in a clinically similar disease, indicating that the two proteins participate in common cellular pathways [83, 95]. Although the primary storage in NPC is cholesterol, other lipids and lipid constituents accumulate in visceral organs and the nervous system, including bis(monoacylglycerol)phosphate, sphingolipids, and GSLs. Curiously in the brain, there is no overall cholesterol storage, despite the accumulation of unesterified cholesterol in the lysosomal membrane; instead, secondary accumulation of sphingolipids, such as sphingomyelin, and GSLs, in particular GM2 and GM3 gangliosides, likely contribute to NPC neuropathogenesis [83, 95].

Most NPC patients present in infancy or childhood, but adult cases have also been reported and are likely underdiagnosed [83, 95]. Visceral symptoms are more commonly observed in patients at a younger age, whereas neurologic and psychiatric signs are apparent in the late stage of the disease and often define the late‐onset groups. Neurological symptoms are also specific to the different age of onset, and range from delayed developmental motor milestones (early infantile period), gait problems, falls, clumsiness, cataplexy (late infantile and juvenile period), to ataxia psychiatric symptoms, and dementia (adult form) [83, 95].

Molecular pathways that underlie NPC pathogenesis have been studied using various experimental systems, including cat, mouse, zebrafish, fruit fly, nematode and yeast models, or NPC1‐deficient cultured cells [96]. Many of these studies have focused on how altered cholesterol and sphingolipid concentration in membranes affect normal cell physiology. For example, primary cells derived from NPC patients were shown to accumulate not only cholesterol but also sphingosine in the lysosomal membrane [97]. The latter increases Ca^2+^ efflux from the lysosomal compartment, which affects late endosome/lysosome fusion and lysosomal trafficking.

Recently, attention has been given to pathogenic pathways occurring at ER‐lysosome MCSs [48, 49]. Most studies in *NPC1* ^*−/−*^ cells have attributed accumulation of lysosomal cholesterol to impaired cholesterol transport at MCSs. Under normal conditions, NPC1 populates ER‐lysosome MCSs, where it interacts with the sterol transport protein, GRAMD1b, anchored to the ER membrane, and transfers cholesterol from the lysosome to the ER [48]. These authors found that in NPC1‐deficient cells, GRAMD1b is unable to egress cholesterol out of the lysosomes in the absence of NPC1, indicating that the connection between these proteins at ER‐lysosome MCSs is needed for this process. On the other hand, cholesterol accumulation in the lysosomal membrane of NPC1 deficient cells could be rescued by overexpression of ORP1L, through its capacity to promote the formation of ER‐lysosome MCSs rather than its cholesterol transport properties [48]. This implies that a secondary mechanism of cholesterol egress exists at these MCSs.

A concurrent publication showed that ER‐lysosome MCSs, via their control of cholesterol gradients, play an important role in the chronic activation of mTORC1 in NPC1‐deficient cells [49]. In this case, VAP‐OSBP interaction facilitates the transfer of an ER pool of cholesterol to the lysosomes, a required step for activating Rag GTPase and for initiating mTORC1 signaling. In cells lacking NPC1, increased levels of VAP‐OSBP complexes promote the constitutive activation of mTORC1 [49]. This appeared to be due to increased cholesterol shuttling through VAP‐OSBP complexes from the ER to the lysosomal membrane. Increased mTORC1 signaling leads to inhibition of autophagy, with deleterious consequences for cell homeostasis [49].

Both these studies have looked at the number of tethering events between the ER and the endo‐lysosomes in *NPC1* ^*−/−*^ cells by colocalizing fluorescently labeled organellar markers [48, 49]. However, while the ER marker was the same (Sec61b) in both studies, lysosomes were labeled with lysotracker in one and with TMEM192 (transmembrane protein 192), marking a restricted pool of lysosomes, in the other. By counting TMEM192^+^ lysosomes juxtaposed to ER membranes, it was concluded that NPC1 deficiency increases the formation of ER‐lysosome MCSs, which consequently leads to increased mTORC1 activation [49]. On the contrary, lower numbers of ER‐lysosome MCSs were detected in *NPC1* ^*−/−*^ cells co‐labeled with Sec61b and lysotracker, suggesting less cholesterol egress out of the lysosome into the ER [48]. These seemingly contrasting results may just be due to the limited number of TMEM192^+^ lysosomes examined that might have increased the selectivity of the organelles engaged in contact sites, vs the large number of lysotracker^+^ late endosomes/lysosomes that might have diluted the overall estimate of tethering events. In order to address the neurodegenerative aspects of NPC, it would be important to understand the effects of accumulation not only of cholesterol in ER‐lysosome MCSs, but also of gangliosides. If these GSLs cluster within these and other MCSs of neurons, they might drastically alter their structural and functional characteristics, similarly to what has been observed in GM1‐gangliosidosis.

## GD and mito‐lyso contact sites

Gaucher disease, the most common LSD, is caused by deficiency of lysosomal β‐glucocerebrosidase (GCase), a glycosidase encoded by the *GBA1* gene [83]. This LSD is a prototypical glycosphingolipidosis, associated with primary lysosomal accumulation of glucosylceramide (GlcCer), followed by secondary accumulation of glucosylsphingosine and other GSLs, including gangliosides. The primary affected cells in patients with GD are the mononuclear phagocytes, in particular macrophages, often referred to as GD cells, whose foamy appearance is emblematic and diagnostic of the disease. Studies that addressed the hematologic deficiencies and ensuing chronic inflammation in patient‐derived macrophages have linked defects in the autophagy pathway to GSL accumulation [83].

Gaucher disease patients are classified into three main types: type 1, which is non‐neuropathic, and type 2 and 3, which are characterized by early‐onset neurological involvement, progressive death of neurons and glia, microglia activation and neuroinflammation [83]. Besides the severe visceral involvement associated with enlargement of the spleen and liver, neuropathic patients, particularly the chronic type 3 types, who live into adulthood, develop seizures, eye movement defects, incoordination, cognitive deficit, and mental retardation.

Several genetically engineered murine models of GD have been generated and well characterized [83]. These models have been instrumental for dissecting the mechanisms of neuropathogenesis elicited by dying neurons and glia, which include altered processing and release of cytokines and chemokine, defective immune and inflammatory responses with activation of the inflammasome, as well as chronic activation of the complement cascade [83].

More than a decade ago, it was discovered that mutations in *GBA1* were associated with increased incidence of PD, in both GD type 1 non‐neuropathic patients and carriers [98, 99]. It was later demonstrated in large population genetic studies that sporadic PD patients have a high incidence of *GBA1* as well as other lysosomal gene mutations, findings that underscored the involvement of the lysosomal degradative system and, in particular, GCase deficiency in the pathogenesis of the disease [98]. It is now well established that *GBA1* mutations represent the highest genetic risk factor for the development of PD and other forms of dementia with Lewy bodies [98]. These adult neurodegenerative conditions are associated with aggregation of α‐synuclein in neurons of the *substantia nigra*, the hippocampus and cerebral cortex. It is noteworthy that GCase levels seem to inversely correlate with α‐synuclein aggregation in both *in vitro* and *in vivo* model systems [98]. However, the potential interaction between these molecules is still unclear, although GlcCer has been proposed to change the conformation of α‐synuclein, mediating the assembly of these toxic protein aggregates [100].

Studies on the neuropathic forms of GD have primarily focused on the molecular consequences of lysosomal dysfunction as a result of GlcCer accumulation. However, cellular pathways involving other organelles, such as activation of the ER stress response and impaired mitophagy, have also been implicated in GD pathogenesis. Therefore, it is possible that the effects of membrane accumulation of GlcCer go beyond disruption of lysosomal integrity and involve other organelles (i.e., ER and mitochondria) likely through MCSs. Indeed, recent studies have linked *GBA1* mutations with dysregulation of mito‐lyso contact sites. PD patients iPSC‐derived neurons with *GBA1* mutations have decreased levels of the Rab7‐GTPase activating protein, TBC1D15 preventing the untethering of mito‐lyso MCSs [64]. Although the molecular mechanisms have not been fully dissected, increased levels of GlcCer seem to be responsible for loss of TBC1D15 untethering activity and prolonged mito‐lyso contact duration [64]. Considering that mito‐lyso MCSs have been implicated in the movement of mitochondria along the axons, increased tethering between the two organelles hampers the timely positioning of axonal mitochondria, resulting in defective mitochondrial respiration [64]. Interestingly, wild‐type iPSCs treated with exogenous GlcCer, or with the GCase inhibitor conduritol‐B epoxide (CBE), commonly used as chemical inducer of a GD phenotype [101], show similar increment in mito‐lyso tethering events [64]. These results suggest a direct involvement of these MCSs in mitochondrial positioning downstream of GlcCer accumulation. Although not proven, it is conceivable that this pathogenic mechanism may underlie not only PD, but also GD, given that GlcCer accumulation is the primary contributor of disease progression.

## Conclusions and challenges ahead

Through assembly and disassembly of MCSs between organelles and the PM, eukaryotic cells can fine‐tune the variety and amplitude of metabolic signaling events to maximize their functions. These dynamic interconnections among all organellar membranes and the PM suggest that many cellular functions involving membranes occur at sites where organelles tether. As key components of MCSs, lipids, such as GSLs, play a central role in dictating MCS composition and, in turn, the pathways they govern. This is particularly relevant for the brain, which has one of the highest lipid contents among all organs. It is therefore not surprising that alteration of lipid homeostasis, particularly GSLs, in neuronal MCSs, has been linked to neuropathogenesis in many adult and pediatric neurodegenerative diseases. This concept has been demonstrated in just a few pediatric, neurodegenerative LSDs associated with abnormal catabolism of GSLs, but this number is likely to increase as investigators are learning more about the molecular pathways happening at MCSs. However, many questions still remain unanswered. For instance: what other molecular tethers or MCSs residents exist in different contact sites?, how are these molecules influenced by altered GSLs' levels and distribution?, what are the mechanisms by which changes in GSLs' concentration, due to primary or secondary lysosomal defects, translate into alterations of normal physiological processes at MCSs?, which molecules are involved in shuttling GSLs across MCSs?, and how are they recruited and regulated? Addressing these questions will begin to uncover the functional roles of GSLs in shaping the properties of MCSs in health and disease.

## Conflict of interest

The authors declare no conflict of interest.

## Author contributions

Ad'A conceived and designed the outline of this review. JAW, IA wrote the first draft of the manuscript and reviewed the literature. JAW and Ad'A wrote and edited the manuscript. DvdV created and edited the figure and contributed to reviewing the literature and editing of the manuscript.

## Data Availability

There are no data to report, as this is a review.
